# Draft Genome Sequence of Bacillus thuringiensis DPC6431, Producer of the Bacteriocin Thuricin CD

**DOI:** 10.1128/MRA.00398-19

**Published:** 2019-08-15

**Authors:** Sara Arbulu, Kiera Murphy, Orla O’Sullivan, Mary C. Rea, Colin Hill, R. Paul Ross

**Affiliations:** a Food Biosciences Department, Teagasc Food Research Centre, Fermoy, County Cork, Ireland; b APC Microbiome Ireland, University College Cork, Cork, Ireland; c School of Microbiology, University College Cork, Cork, Ireland; Loyola University Chicago

## Abstract

We report the draft genome sequence of Bacillus thuringiensis DPC6431, a producer of the anticlostridial bacteriocin thuricin CD and isolated from a human fecal sample. The assembly comprises 96 contigs for a total of 5,581,839 bp, with 32.5% G+C content.

## ANNOUNCEMENT

Bacillus spp. are ubiquitous Gram-positive and spore-forming bacteria known to produce ribosomally and nonribosomally synthesized antimicrobials, as well as enzymes and other metabolites with biotechnological potential (1, 2). Bacillus thuringiensis DPC6431 was isolated from a human fecal sample and produces the two-component sactibiotic thuricin CD, a narrow-spectrum bacteriocin which is particularly active against Clostridium difficile (3). In order to isolate the strain, stool samples were diluted in anaerobic diluent and spread on Wilkins-Chalgren anaerobic plates (Oxoid). Colonies were overlaid with *Clostridium* agar (Oxoid) inoculated with C. difficile ATCC 43593. Inhibition halos led to the detection of B. thuringiensis DPC6431 that was further purified and grown in brain heart infusion broth (BHI; Oxoid). According to biochemical tests with the API 50CHB and 20E kits (bioMérieux), the isolate was classified as a *Bacillus* sp. Further sequencing of the 16S rRNA and *gyrB* genes determined the isolate to belong to B. thuringiensis. Thuricin CD was purified from cell-free supernatants of BHI broth B. thuringiensis DPC6431 cultures, and molecular mass and structure were confirmed by matrix-assisted laser desorption ionization–time of flight (MALDI-TOF) mass spectrometry and nuclear magnetic resonance (NMR), respectively. Genetic sequencing of the thuricin CD operon was also performed (3). Thuricin CD has shown an effect on C. difficile similar to that of traditional antibiotics, and it is active against biofilm formation (4, 5). Moreover, it displayed minimal impact on a human gut microbiota research model (6), and preliminary trials for oral and rectal administration of thuricin CD in mice and pigs, respectively, have also been conducted (7).

The strain is designated B. thuringiensis DPC6431 (from the Dairy Products Centre Culture Collection) and APC20 (from the APC Microbiome Ireland culture collection). The strain is also deposited with the National Collection of Industrial and Marine Bacteria (accession number 41490).

B. thuringiensis DPC6431 was grown overnight on agar plates BHI at 37°C from the glycerol −80°C stocks and then transferred to BHI broth for DNA extraction. The genomic DNA of B. thuringiensis DPC6431 was isolated using a DNeasy UltraClean microbial kit (Qiagen) and assessed using a NanoDrop ND-1000 spectrophotometer and by electrophoresis. Genomic DNA libraries were prepared using the Nextera XT library prep kit (Illumina, San Diego, CA, USA), following the manufacturer’s protocol but with the following modifications: 2 ng of DNA instead of 1 ng was used as input, and the PCR elongation time was increased to 1 min from 30 s. DNA quantification and library preparation were carried out on a Hamilton Microlab Star automated liquid-handling system. Pooled libraries were quantified using the Kapa Biosystems library quantification kit for Illumina on a Roche LightCycler 96 quantitative PCR (qPCR) machine. Libraries were sequenced on an Illumina HiSeq platform using a 250-bp paired-end protocol. Reads were adapter trimmed using Trimmomatic 0.30 with a sliding window quality cutoff of Q15 (8). *De novo* assembly was performed on samples using SPAdes version 3.7 (9), and contigs were annotated using the RAST server (10–12). Both platforms were used on default mode.

The whole-genome sequencing was performed at 30× coverage, yielding a total of 1,824,237 reads after trimming and quality filtering. The draft genome of B. thuringiensis DPC6431 consists of 96 contigs with a contig *N*_50_ length of 304,462 bp, a total of 5,581,839 bp, and a G+C content of 32.5%. The largest contig was 756,911 bp, and the smallest contig was 255 bp. The median insert size was 855 bases. The total number of coding sequences was 5,894, and the number of RNAs was 107.

*In silico* analysis using the BAGEL4 Web server (13) confirmed the presence of a thuricin CD biosynthetic cluster and revealed the presence of cerein B structural, transporter, regulation, and immunity genes, as well as other areas of interest related to linear azol(in)e-containing peptides (LAPs). Two potential clustered regularly interspaced short palindromic repeat (CRISPR) arrays involved in antiviral defense mechanisms were identified using CRISPRFinder (14). The public availability of this draft genome will strengthen the value of the strain as a bacteriocin producer and allow for deeper analysis for future biotechnological applications.

### Data availability.

This draft genome sequence has been deposited at DDBJ/ENA/GenBank under the accession number SCLP00000000. The version described in this paper is version SCLP01000000. Raw sequencing data have been deposited in the SRA database under accession number PRJNA515439.
